# Telepractice with Preschool Children: Speech-Language Pathologists' Perspectives in Turkey

**DOI:** 10.5195/ijt.2022.6465

**Published:** 2022-12-13

**Authors:** Merve Dilbaz Gürsoy, Tuğçe Karahan Tığrak, Ayşen Köse

**Affiliations:** Department of Speech and Language Therapy, Faculty of Health Sciences, Hacettepe University, Ankara, Turkey

**Keywords:** Preschool children, Speech-language pathology, Telepractice, Turkey

## Abstract

The purpose of this research was to investigate speech-language pathologists' (SLPs) perspectives, attitudes, and experiences of using telepractice for preschoolers in Turkey. A mixed-method online survey was used with SLPs who implemented telepractice with preschool children. Frequency distribution and theme analysis were used to examine the data. Therapy was the most offered online service (98%). Further, 67% of SLPs worked with speech sound disorders. More than half of SLPs felt confident offering telepractice to preschoolers. Most respondents thought that telepractice was an appropriate and easily accessible approach for preschool children, with the applicability of telepractice connected to a child's type of problem. The SLPs were motivated by the numerous advantages of telepractice. However, their opinions were divided when telepractice was compared to in-person treatment. The SLPs in Turkey must be better educated about telepractice, and clinical standards established. The findings point to areas of telepractice that might be improved for preschoolers, especially in Turkey.

The American Speech-Language-Hearing Association (ASHA) regards telepractice as the application of telecommunications technology for assessment, intervention, and consultation in speech-language pathology professional services (ASHA, 2016). According to ASHA, the use of telepractice must be equivalent to the quality of in-person services and consistent with adherence to ASHA standards of practice. ASHA (2021) provides guidance on SLP roles and responsibilities.

According to a definition provided by the World Health Organization (WHO), telepractice is the “delivery of health care services where patients and health care providers are separated by distance” (WHO, 2021). The term is variously referred to as “telehealth,” “teletherapy,” or “telepractice” in the literature (Ferguson et al., 2019; May & Erickson, 2014). In this study, the term “telepractice” is used to include all.

Telepractice has both advantages and disadvantages when compared to in-person speech and language therapy services. Studies have provided evidence that telepractice is a feasible, effective, and appropriate model for providing SLP services to a broad range of patients (ASHA, 2021; Hill & Theodoros, 2002; Mashima & Doarn, 2008). Among the advantages of telepractice is it can be used for any age group or any speech and language disorder (Costanzo et al., 2020; Ferguson et al., 2019; Weidner & Lowman, 2020). Another well-known advantage of telepractice is eliminating geographic barriers and physical barriers (De Araújo Novaes, 2020; Edwards et al., 2012). Telepractice makes it easier to obtain SLP services for individuals who need them and have to travel because of distance. Also, telepractice facilitates attendance at SLP sessions for patients who have a physical barrier to attending in-person therapy (Edwards et al., 2012). Another advantage of telepractice is early diagnosis (De Araújo Novaes, 2020), saving time and costs (Mashima & Doarn, 2008). Early intervention can reduce the negative effects of speech and language disorders in childhood. Some of the disadvantages of telepractice are: limited access to adequate technology, internet connection problems, lack of training, and lack of suitable assessment and therapy resources for telepractice (Aggarwal et al., 2020; Hill & Theodoros, 2002; Mashima & Doarn, 2008; May & Erickson, 2014).

Children with speech and language disorders in rural and remote areas may be at a disadvantage because of poor access to SLP services (Mashima & Doarn, 2008). Clinicians in different countries have researched the effectiveness of using telepractice to diagnose, assess, and treat individuals with communication, language, and swallowing disorders who otherwise might not have access to SLP services. It can be difficult in some cities for patients to find a speech-language pathologist, especially for countries that have developing SLP services, such as Turkey. Since the last decade, there has been an undergraduate speech and language therapy program in Turkey (YOK, 2022). Thus, the previously inadequate number of speech and language therapists has expanded, and the demand for specialists in this domain has grown throughout the country.

Several occupations, including SLPs, have been negatively affected by the COVID-19 lockdown and social distancing. Some SLP services require in-person communication between the SLPs and children, as well as their parents, especially if SLPs need to touch clients for assessment and/or treatment. It was difficult to maintain in-person treatment during the COVID-19 pandemic and lockdown periods (Tohidast et al., 2020). Yet, children with speech and language disorders need SLP services for several reasons, such as critical age, comorbidities with speech and language disorders, and later effects in childhood. Consequently, given the need for continuing therapy sessions during the COVID-19 pandemic, SLP services in many countries rapidly converted from in-person to online delivery (Law et al., 2021); telepractice may continue to be offered for some time. According to a review of the literature, SLP telepractice increased due to COVID-19 (ASHA, 2021).

To meet the individualized needs of clients, many SLPs in Turkey had to adapt their assessment and intervention programs to telepractice. Experience gained during the pandemic contributed to improving the telepractice. Thus, many clients who did not have the opportunity to attend SLP assessment and intervention benefited from telepractice regularly and on time. Using telepractice to provide SLP services for children with speech and language disorders is a high-quality alternative during challenges such as a pandemic.

## Telepractice for Children

Because of the importance of early intervention, telepractice is of value for preschool children due to factors such as critical age, the correlation of speech and language problems with other areas of development, and the later effect on children.

There is growing evidence that telepractice is as effective as in-person service delivery for pediatric assessment. According to the findings of a pilot research study conducted in Australia (Waite et al., 2006) an internet-based assessment protocol provided through video teleconferencing has the potential to be a clinically reliable technique for assessing pediatric speech disorders. Clinicians were able to observe the child's communication environment unobtrusively and provide guidance to parents.

Telepractice is also as effective as in-person pediatric service delivery for intervention. Several studies demonstrated that telepractice intervention with children with autism spectrum disorder has successful outcomes (Hao et al., 2020; Sutherland, et al. 2018). Hao et al. (2020) compared the efficacy of teletherapy and in-person therapy parent-based intervention for children an average of 5 years old with autism spectrum disorders. The authors reported significant improvement in children's lexical diversity and morphosyntactic complexity, and parental fidelity, regardless of the use of teletherapy or in-person therapy.

Telepractice is also effective for children with speech sound disorders (Coufal et al., 2018). A systematic review study by Wales et al. (2017) examined the effectiveness of intervention delivered via telepractice for primary school-age children with speech and/or language difficulties. Findings revealed that children who had speech and language therapy using telepractice made similar improvements.

Mashima and Doarn (2008) demonstrated that telepractice is a viable treatment option for children with special needs and can be used to support the delivery of speech-language therapy services in schools. Coufal et al. (2018) compared traditional therapy and telepractice for children aged 6.0 to 9.5 years old who received speech therapy for speech sound production with no co-occurring communication disorder. They found no statistically significant differences between the two treatment groups, evidence that telepractice use with school-age children who have speech sound disorders is equivalent to traditional therapy.

More recently, the results of telepractice intervention with children with velopharyngeal insufficiency and compensatory articulation after cleft palate repair were reported by Pamplona and Ysunza (2020). The children with cleft palate had significant improvement in the severity of compensatory articulation after one month of two 45 minutes weekly telepractice sessions.

There is promising evidence to support telepractice treatment of stuttering in preschool children through parent counseling. Direct therapy with telepractice has also been shown to be effective for children with fluency disorder (Edwards et al., 2012; Lowe et al., 2013; McGill et al., 2019). McGill et al. (2019) included seven studies that reported stuttering treatment via telepractice using the Lidcombe Program, Camperdown Program, and integrated treatment protocols into their review. While the results of telepractice treatment for adults who stutter were generally positive, there is even more evidence for the successful treatment of stuttering in young children (Lowe et al., 2013).

Surveys were conducted on the perspectives and applications of SLPs on telepractice in different countries. According to a survey by ASHA (2016) with participation by 476 SLPs, school-age children were the age group most served by telepractice, followed by preschool children. Most of the SLPs used telepractice for treatment (96.4%). The most common group receiving telepractice were clients with language disorders (73.3%), followed by articulation/phonological disorders (70.5%)(ASHA, 2016). A survey investigation of SLPs in India demonstrated that SLPs primarily served the pediatric population (85%), and also language disorders in childhood (75%) followed by fluency disorder (45.5%) (Aggarwal et al., 2020). In Australia, a survey of SLPs revealed that telepractice is mostly used for direct expressive language therapy with children (Hill & Miller, 2012). Similar results were also published by Fong et al. (2021) in Hong Kong. SLP attitudes, barriers, and benefits, as well as reasons for using telepractice, have also been highlighted in studies (Tucker, 2012).

A goal for the future is for telepractice services to be easily integrated into SLPs' routine clinical responsibilities. First and foremost, accessibility to telepractice and the needs of SLPs for the effective advancement of remote therapy services are critical to discern.

## Aim of the Study

The main purpose of this study was to investigate the thoughts, attitudes, and experiences of SLPs working with preschoolers in Turkey regarding telepractice. There is a lack of information about telepractice for both professionals and clients in Turkey. It is crucial to know about SLPs' perceptions of telepractice as a service delivery approach in preschool children, as well as its benefits and challenges.

Although telepractice studies are increasing in recent years, there is a need for time and resources about different age groups and disorder types. As far as the authors know, there is no study that examines the perspectives of SLPs on the telepractice processes of preschool children with communication disorders. This is surprising since numerous studies, including ASHA's study (ASHA, 2016), show that children are the most served clinical age group via telepractice. One research study had the greatest number of preschool children (Kraljević et al., 2020).

## Method

A cross-sectional, mixed-method online survey design was used aiming to reveal the perspective of SLPs who are working with preschool children via telepractice. This study combined qualitative findings regarded from open-ended questions which asked about advantages and disadvantages of telepractice, and quantitative results from closed-ended questions in the survey. The researchers determined the themes and their sub-themes by examining the answers to the open-ended questions. The frequency distributions were established by analysing the responses to the closed-ended questions.

### Survey Design and Procedures

This study protocol was approved by the Hacettepe University Ethics Commission. A pilot survey was developed by the investigators based on the Burns et al. (2008) guidelines. Questions in the survey were designed according to previous literature of similar SLP perspective surveys. Initially, this pilot survey was sent by e-mail to seven experienced SLPs and one linguist for their expert opinion (3 M; 5 F; 2 Ph.D.; 4 MSc; 2 Bachelor's degree). These SLPs and a linguist were asked to rate the items of the survey as relevant; relevant but needing to be corrected; and not relevant. This process was completed between December 8–20, 2020. None of the questions were rated as not relevant by the experts, though the language of some questions needed to be improved for clarity. As a result of expert opinions, two questions were added to the survey. A revised version of the survey was created online using Google forms; the online form was e-mailed to the other eight SLPs for the pilot testing. All testers confirmed the survey contained extensive and understandable questions. Following the completion of pilot testing, some technical revisions were made to the online survey.

The final version of the survey contained 34 items, including five open-ended questions and 29 closed questions, of which 11 were Likert-type questions. Open-ended questions related to some demographic information about SLPs (i.e., age and home-city), advantages and disadvantages of telepractice, and the number of preschool clients who had intervention via telepractice. Closed question formats were multiple-choice, yes-no options, and 5-point Likert scales (1=strongly disagree, 5=strongly agree). Closed questions were based on demographic details, practice experience of telepractice (e.g., types of caseload, telepractice platforms, number of sessions per week, duration of a session, self-reliance in telepractice), attitudes, and beliefs related to telepractice. The online survey was arranged so that participants could proceed based on their responses. SLPs who had no telepractice experience with preschoolers did not receive all items.

The online Google form survey was used for the data collection. Data collection was completed in one month, after which the survey link was closed. The link to the online survey was shared via e-mail, social media applications, and WhatsApp. In this way, the target number of participants was reached. The survey required 10–15 minutes for completion.

### Participants

Participants were required to meet the following inclusion criteria: (a) currently working as a SLP for at least 18 months in Turkey, and (b) prior experience implementing telepractice with preschool children. At the beginning of the online survey, participants were provided with an informed consent form. Participants could proceed to the survey questions only after they completed the consent form.

### Data Analysis

The research included both quantitative and qualitative data. Descriptive statistics were used to summarize continuous variables, while discrete variables were summarized using frequency and percentage. The responses obtained in the study were analysed using SPSS. Quantitative data was analysed through frequency distribution percentages. Thematic analysis was used to examine qualitative data. Coding was utilized with the two researchers conducting all aspects of the analysis. Together these researchers reviewed all responses and the entire coding process to ensure accuracy. To avoid inconsistency, the same two researchers discussed disagreements until a consensus was reached.

## Results

A total of 147 SLPs responded to the survey, of which 42 met the criteria of the study. The remaining 105 participants were excluded from the study due to the following reasons: (a) 41 had not experienced telepractice, (b) 45 had been working as an SLP less than 18 months, and (c) 19 had not experienced telepractice with preschoolers. Analyses were performed on the responses of 42 SLPs. The study's data was examined quantitatively and qualitatively.

The majority of the participants were female, with an average age of approximately 30 years (range: 23–46 years, *SD*: 6.15). While most of the participants (88%) lived in various metropolitan cities in different regions of Turkey, 12% lived in small cities. The demographic information of the participants is shown in Table 1. Only a small number of participants (5%) had used telepractice with preschoolers before the COVID-19 pandemic, and most participants (93%) indicated that their telepractice services increased with the pandemic.

**Table 1:** Demographics of Participants

Variables	Experienced in Telepractice with preschoolers n = 42 (%)
Gender	
Male	6 (14)
Female	36 (86)
Education	
Bachelor degree	19 (45)
M.Sc	19 (45)
Ph.D.	4 (10)
Years of clinical experience	
18 months – 3 years	16 (38)
3 – 5 years	9 (21)
5 – 10 years	13 (31)
>10 years	4 (10)
Work setting^*^	
Public hospital	1 (2)
Private hospital	2 (5)
University	8 (19)
Private clinic	19 (45)
Special education and rehabilitation center	15 (36)

*Note*.

* Total >100% as some participants reported multiple work settings.

### Quantitative Data

#### Information Sources About Telepractice

**Figure 1:**
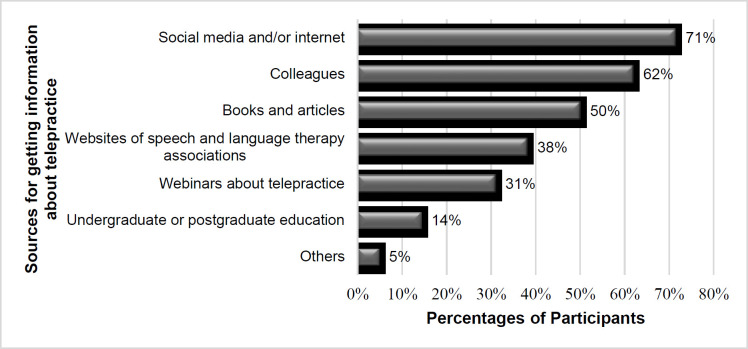
Sources for Obtaining Information about Telepractice

The participants were asked whether they had taken a course about telepractice or had a chance to experience telepractice in their speech-language pathology education. Most did not take such a course (93%) and did not have any opportunity to practice telepractice in their education (90%). SLPs learned about telepractice from different sources such as the internet, books, and articles. The majority reported that the most frequent source of learning about telepractice was social media and/or the internet (71%), followed by colleagues (62%) (Figure 1).

### Service Delivery to Preschoolers via Telepractice

**Figure 2:**
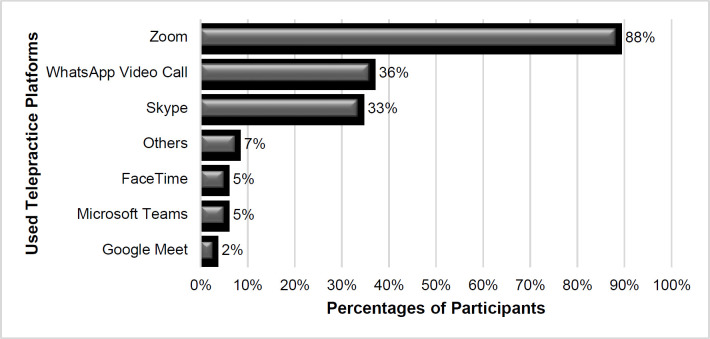
Platforms Used for Telepractice

The SLPs reported that they mostly use telepractice for intervention (98%). While 74% used telepractice for assessment and counseling, only 26% used telepractice for screening. The SLPs were asked which applications or programs were used for telepractice. There were several platforms employed: the Zoom application was used by most (88%), followed by WhatsApp video calls (36%), and Skype (33%) (Figure 2). The SLPs were asked to report how often they met with preschool clients in telepractice; only 2% said more than two times a week, 5% reported once every two weeks, 14% said two times a week, and 79% reported once a week. The telepractice session durations of SLPs with preschoolers were usually 30–45 minutes (55%), followed by 45–60 minutes (26%), 20–30 minutes (17%), or more than 60 minutes (2%) respectively.

**Figure 3:**
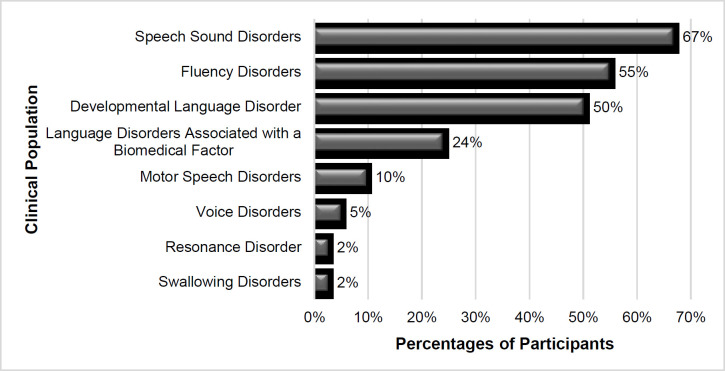
Distribution of Disorders to Whom Telepractice Services Were Delivered to Preschoolers in Turkey

The SLPs were asked how many preschoolers had finished speech and language therapy through telepractice. The 42 SLPs using telepractice conducted a service delivery average of 3 preschoolers. As far as the age groups of caseloads, the majority of SLPs reported that the most common age group was preschool children (86%), followed by school-age children (62%), adults (36%), adolescents (33%), and elders (10%). These SLPs also reported the distribution of disorders among preschoolers to whom they delivered service via telepractice. The most common disorder was speech sound disorder (67%), followed by fluency disorder (55%) (Figure 3).

### Attitudes Towards Telepractice with Preschoolers

The SLPs' perceptions about telepractice were also addressed in our questions. All 42 SLPs were asked to rate their agreement level with the 11 statements about telepractice (Table 2). Most SLPs (59,5%) stated that telepractice made it easier for people to access speech-language pathology services, and 40% would increase the use of telepractice in the future. More than 50% of SLPs remarked that telepractice requires standard clinical protocols. The applicability of telepractice with preschool children, according to 78% of the SLPs, is related to the child's type of disorder.

**Table 2:** Attitudes about Telepractice Held by SLPs in Turkey

Telepractice Statement	Strongly disagree	Disagree	Unsure	Agree	Strongly agree
	n (%)	n (%)	n (%)	n (%)	n (%)
Standard clinical protocols are required for telepractice.	2 (4,8)	5 (11,9)	9 (21,4)	14 (33,3)	12 (28,6)
The institution where I work supports telepractice (financial opportunities, technical facilities, training, etc.).	3 (7,1)	4 (9,5)	6 (14,3)	13 (31,0)	16 (38,1)
I think that the preschool children who had an intervention with telepractice progress as much as face-to-face therapy.	1 (2,4)	10 (23,8)	12 (28,6)	15 (35,7)	4 (9,5)
I think that telepractice with preschool children is equally efficient and effective as face-to-face therapy.	3 (7,1)	14 (33,3)	11 (26,2)	12 (28,6)	2 (4,8)
I think that the therapeutic relationship with preschool children in telepractice is ensured.	3 (7,1)	8 (19,0)	14 (33,3)	12 (28,6)	5 (11,9)
I think that the therapeutic relationship with preschool children in telepractice is ensured such as in face-to-face therapy.	2 (4,8)	16 (38,1)	13 (31,0)	8 (19,0)	3 (7,1)
I think that the telepractice relationship with preschool children, the therapeutic relationship with family and teachers is ensured such as in face-to-face therapy.	2 (4,8)	8 (19,0)	12 (28,6)	13 (31,0)	7 (16,7)
The applicability of telepractice with preschool children is related to the type of disorder the child has.	2 (4,8)	3 (7,1)	4 (9,5)	17 (40,5)	16 (38,1)
I recommend using telepractice with preschool children.	1 (2,4)	5 (11,9)	11 (26,2)	15 (35,7)	10 (23,8)
I think that telepractice makes it easier for individuals to access speech-language pathology services.	0,0	0,0	3 (7,1)	14 (33,3)	25 (59,5)
I think that I will use telepractice more in my future professional life.	0,0	3 (7,1)	8 (19,0)	14 (33,3)	17 (40,5)

**Figure 4:**
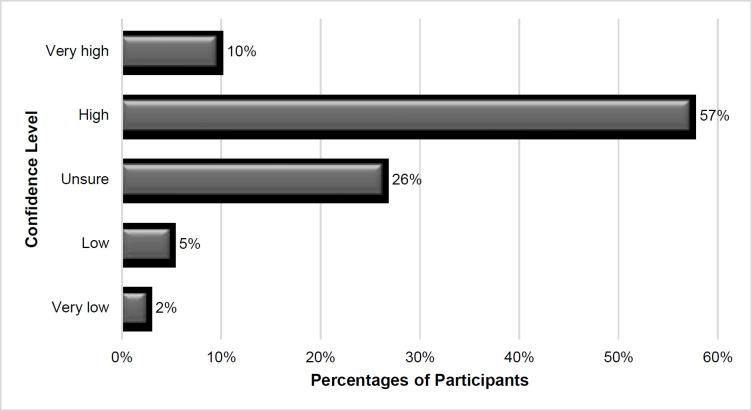
The SLPs' Confidence in Telepractice with Preschoolers in Turkey

More than half of the SLPs indicated that they felt confident in providing telepractice with preschool children (Figure 4). That the remaining SLPs do not feel confident providing telepractice is perhaps a result of the difficulties they face in teletherapy. Of the participants, 88% stated that they had the most difficulty in the application of intervention that requires in-person contact. Other difficulties included lack of appropriate assessment and therapy materials for telepractice (62%), network problems (57%), accessibility of clients to technology and the internet (48%), and lack of knowledge about technology (43%).

## Qualitative Data

There were two open-ended questions in the survey related to benefits and barriers in telepractice speech-language pathology services.

### Benefits of Telepractice Services

The SLPs related some perceived benefits of telepractice. Five themes emerged. The first theme, “accessibility of speech-language pathology service delivery,” emerged from the responses that telepractice provides the SLP service delivery opportunities for clients with distance or physical barriers, and also easier access for all participants. The second theme was “positive differences that telepractice made” which emerged from the participants' responses about time, flexibility, cost-benefit, and comfort factors. A third, expected theme, “COVID-19 and health,” emerged because this study was conducted during the COVID-19 pandemic period; participants assessed telepractice as a safe option. In addition, the participants indicated that children in a critical period could get SLP assessment and intervention during pandemic times. A fourth theme was “technology and the internet,” which is about the advantages of technology and that children are accustomed to using electronic devices. A fifth theme “efficiency of telepractice services,” accounted for participants' responses towards the specific benefits for children and their therapy progress. See Table 3 for the themes, categories, and supporting quotes for the benefits of telepractice.

**Table 3:** Benefits of Telepractice Perceived by Speech-language Pathologists in Turkey

Themes	Subthemes	Supporting quotation
1. Accessibility of speech-language pathology service (n=24)	(1) Clients with distance (n=9)	“Reaching patients in different cities or locations” “It [telepractice] allows us to reach people who do not have speech and language therapy services in their city”
	(2) Clients with physical barrier (n=2)	“It [telepractice] provides the opportunity to reach people with mobility restrictions” “Ideal for those with mobility issues”
	(3) Greater accessibility (n=13)	“Accessibility to therapy is very easy for both patients and therapists.” “Therapy services are becoming more accessible.” “An alternative way for those who do not have a chance to reach a speech and language therapist.”
2. Positive clinical differences that telepractice made (n=38)	(1) Saving on time (n=18)	“It [telepractice] saves time.” “I think it [telepractice] saves time for both the patient and the speech and language therapist.”
	(2) Flexible work hours (n=8)	“… providing therapy at any hour of the day” “More flexible hours of therapy can be provided.”
	(3) Cost-benefit (n=2)	“More economical (time and cost)” “No transportation costs”
	(4) Comfort (n=6)	“More comfortable” “Children are not tired from the travel because they are at home.” “The teletherapy reduces physical fatigue.”
	(5) Reduced travel problem (n=4)	“Reduction of transportation problem” “No transportation problems”
3. COVID-19 and Health (n=16)	(1) COVID-19 (n=13)	“… healthier during the pandemic” “Reducing the risk of Covid-19” “I think it is safe for patients and clinicians, especially during the pandemic period.”
	(2) Critical period of children (n=3)	“It [telepractice] provided intervention to children in the most critical developmental period in the pandemic.” “Continuation of therapy support during the pandemic period”
4. Technology and the Internet (n=9)	(1) Advantages of technology (n=6)	“… provides opportunity to benefit from technology” “I can scan most of the materials on the computer and access them easily.”
	(2) Technology and children (n= 3)	“Children's love of computer games makes therapy more fun.” “I think children who like to look at the screen focus more. The session is not interrupted by material like a board game, the children do not want to play and the session is fuller, we can practice more and progress faster by telepractice”
5. Efficiency of telepractice service (n=9)	(1) Additional benefits for children (n=5)	“The chance to include the family in therapy and to observe in the home environment.” “Parent-based interventions can be provided in teletherapy.” “I think that teletherapy is suitable for both child and parent-centered interventions. The most important point is to set goals that are suitable for the family and the child.”
	(2) Therapy progress (n=4)	“As long as their parents are with them and support the therapy process, I see that their gains in therapy are sufficient, we progress as far as face-to-face therapy in teletherapy.” “Families who can apply and follow directions correctly can be as effective as face-to-face therapies.”

### Barriers of Telepractice Services in Turkey

Six themes emerged according to SLPs' perceptions of the barriers of telepractice. The first theme, “technology as a barrier” has 3 associated categories. The SLPs mostly specified that internet connectivity problems, quality of sound and video, and limited technical knowledge were barriers to using telepractice services. Another theme was “specific problems in children.” Early age, attention/regulation problems on a screen, and additional diagnoses (e.g., autism spectrum disorder, attention deficit hyperactivity disorder) of children were indicated by many participants as barriers. Moreover, some of the participants argued that telepractice is not appropriate for every child. The third theme, “materials suitable for telepractice,” indicated a lack of telepractice materials for assessment and therapy. “Lack of physical contact with the use of telepractice” was distilled as a fourth theme due to lack of in-person communication and limited stimuli in telepractice. Another barrier theme was “time,” which is about sessions' durations, set-up time, and screen time. The last theme of barriers of telepractice, “concerns,” accounted for participants' responses about ethical issues, and whether telepractice is as effective as in-person therapy. See Table 4 for the themes, categories, and supporting quotes for the barriers of telepractice.

**Table 4:** Barriers of Telepractice Perceived by Speech-language Pathologists in Turkey

Themes	Subthemes	Supporting quotation
1. Technology as a barrier (n=27)	(1) Internet connectivity problem (n=18)	“Internet is not stable.” “Poor connection quality.”
	(2) Quality of sound and video (n=5)	“Not being able to give correct feedback in cases where the sound and image quality is low (especially in speech sound disorders) due to my inability to hear the sound well.” “… Camera and microphone quality have a direct impact on communication.”
	(3) Lack of knowledge, confidence (n=4)	“Parents' technology knowledge should be insufficient.” “Sometimes, it may be necessary to start with the most basic “what is Zoom” experiments with the family.”
2. Specific problems in children (n=33)	(1) Early ages (n=10)	“Difficulty in assessment and therapy with young children” “It can be difficult for preschool children to participate in a motivated way.” “It is difficult to maintain the attention of children, especially in the preschool period.”
	(2) Attention & regulation problems (n=15)	“Children are more easily distracted.” “Arranging a child's regulation can be difficult” “The challenge of trying to keep kids in front of a screen.”
	(3) Additional diagnosis (n=3)	“I think it is impossible/very difficult to use it as an option, especially in diagnostic groups that require one-on-one therapy sessions.” “Difficult to understand and limiting attention span for children with developmental delay or ADHD.”
	(4) “Telepractice is not appropriate for every child” (n=5)	“Teletherapy is not suitable for everyone (especially pre-school children or ADHD/ASD population).” “Not every case adapts to teletherapy.”
3. Materials suitable for telepractice (n=6)		“I have trouble finding the materials I want.” “Lack of material.”
4. Lack of physical contact with the use of telepractice (n=19)	(1) Face-to-face communication (n=9)	“Difficulty in reading body language, lack of advantages of face-to-face communication.” “Inability to provide an environment of trust with the child because there is no face-to-face communication.”
	(2) Limited stimuli (n=10)	“Inability to apply situations that require physical contact.” “Since we cannot make tactile intervention in terms of the speed of progress, there may be slowdown, etc.” “In speech sound disorders, I have difficulty trying to isolate that sound. Because I can't touch, children can get confused about where to put their speech sounds.”
5. Time (n=8)	(1) Management of therapy sessions' durations (n=4)	“Difficulty determining the duration of therapy.”
	(2) Required additional time for set up (n=1)	“Telepractice requires a lot of preparation.”
	(3) Screen time (n=3)	“Staying in front of the screen for a long time.”
6. Concerns (n=5)	(1) Ethical issues (n=3)	“There is a need for studies on the ethical aspects of teletherapy and for it to become guiding in practice; … the possibility of becoming vulnerable to abuse should be checked”
	(2) Not adequate as face-to-face therapy (n=2)	“I don't think it [telepractice] is as effective as face-to-face training.” “I think it would be insufficient to continue the entire therapy as teletherapy.”

## Discussion

To summarize, the purpose of the current paper was to investigate the experiences and perspectives of SLPs who recently worked with preschool children via telepractice in Turkey. Remote intervention systems, such as telepractice, play a crucial role in facilitating preschoolers' access to SLP services and in raising awareness of the profession in countries such as Turkey, where SLP services are only beginning to become widespread but cannot reach every corner of the country.

Now that telepractice is widely used and SLPs were forced to adopt new working conditions, we aimed for this study to describe the pereptions of SLPs involved in the screening, evaluation, and/or treatment of preschool children, as related to their implementation of telepractice. Because telepractice evaluation and treatment approaches differ for population-specific traits, it is essential to recognize the challenges for each clinical population, and develop individualized solutions and problem resolutions (Swales et al., 2020). Accordingly, it was considered that learning SLPs' perspectives who treat preschool children with speech and language problems would be beneficial, especially because early intervention is crucial.

Because the survey contained comparison items between in-person therapy and telepractice, we required eligible participants to have worked as SLPs for at least 18 months. Given that this study was conducted in Turkey after the pandemic began, respondents needed prior experience in in-person treatment to make the comparison. The vast majority of participants stated that they started to use telepractice as a result of the COVID-19 pandemic, as was anticipated. The COVID-19 pandemic appears to have encouraged telehealth applications in Turkey, as well as worldwide. Given the need for therapy sessions, SLP services rapidly converted from in-person to online applications to meet the challenges of the COVID-19 pandemic (Law et al., 2021).

We believed it was necessary to investigate therapists' knowledge sources and experiences with telepractice to understand their experiences in a country that is quickly changing toward a previously non-traditional (i.e., the use of telepractice) approach. During their undergraduate or postgraduate studies, only 14% of SLPs stated they had learned about telepractice. Only 7% had taken a telepractice course, and just 10% had done telepractice practice as part of their education. The study revealed that SLPs tried to get information most frequently via social media/internet, their colleagues, and books/articles about telepractice. This result is similar to the findings of Aggarwal et al. (2020). However, there was a difference among the participants in the two studies as to whether telepractice requires a standard clinical protocol. Standard procedures were required by 61,9% of participants in this study, which was lower than the previous study's rate of 94,1% (Aggarwal et al., 2020). The current study's findings suggest that many SLPs are trying to obtain effective and standard information for telepractice with preschool children but are unable to do so. Despite the lack of resources, most participants (83%) expressed self-confidence in the use of telepractice with preschool children. Concurrently, Finch et al. (2020) found that telepractice enhanced the confidence of less experienced SLPs in communication with persons with aphasia. Most SLPs also had relatively less clinical experience in this research. Additionally, SLPs in the literature reported that they learned telepractice via the trial-and-error method (Aggarwal et al., 2020; Tucker, 2012). We did not have a trial-and-error choice in our survey.

More than half the participants stated that their workplace supports telepractice by providing some benefits such as financial or technical conveniences. These tools can be used to improve the efficacy of telepractice SLP services in Turkey. Most participants work in centres where their primary responsibility is to serve patients. Telepractice was used by nearly all (98%) of our participants for treatment. Telepractice was primarily used for therapy rather than evaluation by participants in this survey, which is similar to research from various countries (ASHA, 2016; Fong et al., 2021; Hill & Miller, 2012). In contrast to Hill and Miller's (2021) study, there have been changes in the platforms used for telepractice, with SLPs using more audio and video platforms. As seen by the use of online platforms such as Zoom, Skype, WhatsApp video call in current studies, and also this research, technological improvements have played a significant role in enhancing the use of telepractice (Aggarwal et al., 2020; Del Carmen Pamplona & Ysunza, 2020; Fong et al., 2021). Depending on their patients' requirements and circumstances, more than half of SLPs were using several platforms.

In the current research, the SLPs' caseloads were primarily comprised of pediatric patients, especially preschool children. Most therapists met with preschool children once each week for 30–45 minutes of treatment. Although a similar distribuition for the pediatric population was previously reported in the literature (Aggarwal et al., 2020; ASHA, 2016; Fong et al., 2021; Hill & Miller, 2012; Mohan, Anjum & Rao, 2017), the pediatric population ratio in the current study was greater than for the other studies which reported higher telepractice rates in school-aged children. Though we anticipated that the inclusion of SLPs working with preschool children in this study would have an effect on these rates, it might be that children needed to begin or continue their treatment during the pandemic. Moreover, that the pediatric population is the age group most frequently found in these studies suggests that telepractice is more common for children. Therefore, we believe the current study is crucial to reflect on SLP experience in preschool children in Turkey.

Although the disorder with the highest caseload of SLPs has varied in previous studies, the top three categories remained similar: language disorders, articulation/phonological disorders, and fluency (ASHA, 2016; Fong et al., 2021; Hill & Miller, 2012). When examining the distribution of disorders of preschool children receiving telepractice service in the current study, speech sound disorder, developmental language disorder, and fluency disorder all have high frequencies. Management of swallowing problems with telepractice in preschool children was lower than other disorders, with rates found to be similar in prior studies (ASHA, 2016; Hill & Miller, 2012). However, the use of telepractice for resonance disorders, voice disorders, and motor speech disorders was lower among SLPs in Turkey in comparison to data from the USA (ASHA, 2016).

The majority of participants (78,6%) stated that the applicability of telepractice is dependent on a preschool child's type of disorder. It is important to determine why some speech and language problems are more challenging than others to treat via telepractice, and to develop solutions. Despite some limitations, 59.5% of participants recommended the use of telepractice for preschool children because they believe that telepractice makes it easier for individuals to access SLP services. SLPs in Turkey also perceived that telepractice will be used more in their future professional lives (73.8%). Therefore, it is important for the future to determine the benefits and limitations of telepractice for various client populations, as well as how telepractice is used.

In comparative studies there were no significant differences between children who received in-person therapy and those who received telepractice treatment (Coufal et al., 2018; Grogan-Johnson et al., 2013; Hao et al., 2020). Significant improvement at the end of the telepractice period was reported (Del Carmen Pamplona & Ysunza, 2020; Wales et al., 2017). SLPs in the current research stated that they had achieved progress in therapy, and that preschool children benefit from telepractice as much as they do from in-person therapy services. However, the Turkey cohort did not believe telepractice resulted in comparable therapeutic relationships. The attitude that therapeutic relationships with preschool children are not as strong in telepractice contradicts Hines's (2015) findings. Although SLPs were initially concerned about the therapeutic relationship, comparisons revealed that in-person therapy had similar benefits (Freckmann et al., 2017; Hines et al., 2015). In the current study, perhaps SLPs believed the therapeutic relationship was inadequate even when they made progress with preschool children's cases, because they had been forced by the pandemic to quickly transition from traditional therapy to telepractice. Therefore, they did not have enough time to discover solutions to some of the disadvantages of telepractice. Moreover, it is possible that there was insufficient information on outcomes and efficacy as a result of the rapid shift in therapeutic practice.

SLPs were asked about the advantages of telepractice. They mostly mentioned that telepractice saves time and makes speech-language therapy services more accessible. There was no SLP who disagreed. Telepractice is frequently cited in the literature as a viable option for geographic and physical barriers, as well as providing benefits to patients (De Araújo Novaes, 2020; Edwards et al., 2012). When questioned about the benefits of telepractice, it was inevitable that COVID-19 would come up. Many SLPs stated that telepractice reduces risks during the pandemic, and they were able to continue delivering speech-language pathology services because of telepractice. Considering that regular attendance in the speech-language pathology intervention program is required to achieve the intervention goals and achieve effective results (Del Carmen Pamplona & Ysunza, 2020), telepractice is highly beneficial during difficult times such as pandemics. According to some SLPs, the critical period for intervention in preschoolers was not missed thanks to telepractice. It is well known that early childhood speech and/or language disorders have a negative effect on children's future development. Early intervention has been highly emphasized in the literature for many years, with the recommendation that communication disorders should be treated by SLPs within a treatment program. Different telepractice benefits from the literature were presented in this study. Some SLPs stated that they utilized the technology's benefits as well as its disadvantages.

Because it was believed to be significant, “technology and children” was proposed as a separate subtheme. A few SLPs observed that children benefitted from telepractice because they enjoyed devices like computers, tablets, and phones. This might be a component that SLPs consider when choosing a case for telepractice, or it could be a characteristic of the child that they will use to their advantage.

Telepractice has also been reported to offer additional benefits for children. Seeing the home environment, involving the family, and facilitating family-based interventions are some examples. The benefits of delivering services in the child's natural environment are well documented (Theodoros, 2011). Telepractice provides an opportunity, particularly for early diagnosis and intervention, and can help to prevent the progression of disorders (De Araújo Novaes, 2020). Furthermore, it allows for intervention in early childhood language and/or speech disorders, which is beneficial to children's overall development. Telepractice also enables family-based interventions to be carried out with parents of preschool children.

The majority of the SLPs discussed difficulties that are specific to children, such as attention/regulation problems, early ages, and other diagnoses. SLPs reported that the complexity of the patient's diagnosis and overall clinical picture, as well as chronological age, were influential in selecting patients for telepractice in Croatia (Kraljević et al., 2020). Lack of cooperation/difficulty in getting the child to sit was also mentioned by SLPs in India (Aggarwal et al., 2020). Other barriers highlighted by participants included lack of in-person contact and technological inadequacies (e.g., internet connection problems, audio/video concerns, a lack of appropriate assessment/therapy computer-based tools for telepractice, and inadequate accessibility to technology). These disadvantages are consistent with the literature (Aggarwal et al., 2020; Hill & Theodoros, 2002).

Due to the requirement for touch or role models, SLPs' perceptions of the efficacy of telepractice may be affected in some patient groups. In the qualitative data section, participants reported difficulties adapting telepractice to the intervention of some children with speech sound disorders and voice disorders, particularly in the pre-school age. Therapy techniques that involve physical contact, such as phonetic placement, are seen as difficult during telepractice by a vast majority (88%) of respondents.

Standard clinical criteria/protocols must be established, or better information provided, to render telepractice as high-quality as in-person treatment. Each age group and disorder should have its own set of guidelines. Likewise, tele-ethics should be considered, as some of the participants expressed ethical concerns. In order to identify the standard protocols that have been defined as a necessity, training is necessary (Aggarwal et al., 2020). Before the pandemic, none of the participants had taken a telepractice course or practiced telepractice. In this regard, a course in Turkey's speech-language pathology departments could be required. Training might also take place outside of the university. More than half of the SLPs said they were confident in their ability to provide telepractice to preschoolers. Telepractice is expected to become more popular in the future, according to the participants. Against all odds, the participants said that they would profit from the telepractice approach's benefits while avoiding its challenges.

### Limitations

The present study has limitations that may prevent generalization to all countries. Turkey is a country with a relatively young history of speech-language pathology. Participants were not equally distributed in terms of years of professional experience and work setting. Also, most participants lived in major cities. The survey results might have greater validity to telepractice in Turkey if more of the study participants were from rural or small city settings.

Because the survey was specifically developed for SLPs, no information about parental and patient perceptions is available. The perspective and experiences of patients and their families is an area for future research.

## Conclusions

According to the research outcomes, SLPs in Turkey were generally motivated by the many perceived benefits and potential of telepractice. For example, telepractice makes it easier to reach a SLP during preschool age, when the critical period for therapy is essential.

However, study participants also expressed some doubts about telepractice, as compared to in-person therapy.

The outcomes of this study indicate what aspects of telepractice might be improved in Turkey, with applicability worldwide. These include the need for better education for SLPs and establishing standards for telepractice services. Many important challenges and resource concerns must be resolved to promote the application and sustainability of telepractice in clinical services, so that preschool children can benefit from telepractice.

The current study adds to prior evidence that suggests that telepractice will become a feasible, effective, and appropriate model for providing SLP services to a broad range of patients in the future (ASHA, 2021; Hill & Theodoros, 2002; Mashima & Doarn, 2008).
